# The Effect of Robot-Assisted Gait Training on Locomotor Function and Functional Capability for Daily Activities in Children with Cerebral Palsy: A Single-Blinded, Randomized Cross-Over Trial

**DOI:** 10.3390/brainsci10110801

**Published:** 2020-10-30

**Authors:** Li Hua Jin, Shin-seung Yang, Ja Young Choi, Min Kyun Sohn

**Affiliations:** 1 Department of Rehabilitation Medicine, School of Medicine, Chungnam National University, Daejeon 35015, Korea; kimlihua109@naver.com (L.H.J.); ssyang74@cnu.ac.kr (S.-s.Y.); 2 Department of Rehabilitation Medicine, Chungnam National University Hospital, Daejeon 35015, Korea; cielewme@cnuh.co.kr

**Keywords:** robotic-assisted gait training, cerebral palsy, gross motor function

## Abstract

Purpose: The effectiveness of robot-assisted gait training (RAGT) in children with cerebral palsy (CP), especially in terms of improving the performance of daily activities, remains unclear. Therefore, we aimed to investigate the effectiveness of RAGT in children with CP. Methods: In this single-center, single-blinded, randomized cross-over trial, we enrolled 20 children with CP with Gross Motor Function Classification System (GMFCS) levels II–IV (13 males; age range, 6.75 ± 2.15 years). The participants were randomized into the RAGT/standard care (SC) (*n* = 10) and SC/RAGT/SC sequence groups (*n* = 10). Using a Walkbot-K system, the RAGT program comprised 3 × 30-min sessions/week for 6 weeks with a continued SC program. The SC program comprised 2–4 conventional physiotherapy sessions/week for 6 weeks. The Gross Motor Function Measure-88 (GMFM-88), the pediatric functional independence measure (WeeFIM), and the Canadian occupational performance measure (COPM) scores were assessed pre- and post-RAGT or SC periods and treatment, period, follow-up, and carry-over effects were analyzed. Energy expenditure and body composition were measured pre- and post-RAGT. Results: Significant treatment effects were observed in dimensions D and E of the GMFM (D: *p* = 0.018; E: *p* = 0.021) scores, WeeFIM mobility subtotal (*p* = 0.007), and COPM performance (*p* < 0.001) and satisfaction (*p* = 0.001) measure scores. The period, follow-up, and carry-over effects were not statistically significant. The gross energy cost significantly decreased (*p* = 0.041) and the skeletal muscle mass increased (*p* = 0.014) at post-RAGT assessment. The factors associated with functional outcomes showed significant improvements in the GMFM D scores and were mainly observed in children with GMFCS levels II–III compared to those classified at level IV (*p* = 0.038). Conclusion: RAGT had training benefits for children with CP. Specifically, it improved locomotor function and functional capability for daily activities. These effects were better in ambulatory children with CP. However, as SC interventions continued during the RAGT period, these improvements may be also related to multiple treatment effects.

## 1. Introduction

Cerebral palsy (CP) has been reported to be one of the most common causes of motor disabilities in children. CP comprises a group of disorders affecting movement, muscle tone, and posture, resulting in activity limitation due to brain lesions [1]. The severity of the brain lesions restricts a child’s participation in a broad range of life domains [2]. Low participation levels are related to low levels of physical activity and reduced fitness, further reducing mobility and physical activity [3].

Recently, robot-assisted gait training (RAGT) has been used as a repetitive and task-specific therapy for children with CP [4,5]. RAGT has shown promising possibilities to enhance functional outcomes in ambulatory and non-ambulatory children with CP. RAGT involves the practice of complex repetitive gait cycles using body-weight support (BWS) to meet the gait requirements in weak lower limbs and it exerts less cardiorespiratory stress compared to overground gait training or gait training without robot-assistance [6].

In a retrospective analysis involving a large population of children with CP, RAGT improved the 6-min walk test (6MWT) results in all children with CP, whereas Gross Motor Function Measure (GMFM) scores improved only in children classified as Gross Motor Function Classification System (GMFCS) level III [7]. Positive RAGT results have also been reported in terms of improvement in GMFM D (dimension D, standing) or E (dimension E, walking, running, and jumping) scores [8,9,10], gait speed [8,11], and decreased energy costs [12]. However, most reports have involved case studies or retrospective analyses, with only a few randomized controlled trials (RCTs) involving robot training reported in children with CP. Smania et al. [13] found that gait speed and step length improved after 10 robot training sessions. Druzbicki et al. [14] reported that RAGT was not more effective than conventional physiotherapy in terms of gait parameters. Wu et al. [11] reported a significant increase in walking speed and 6MWT results post-RAGT. One recent pragmatic crossover trial found no improvement in walking ability after 5 weeks of RAGT-only intervention [15]. Therefore, the effects of RAGT in children with CP remain unclear.

Walkbot-K (Walkbot-K, P&S Mechanics, Seoul, Korea) is a treadmill-based exoskeletal RAGT system designed to provide intensive training with minimal labor or physical stress to the therapist. The validity of the neuromechanical data and reliability of the test-retest kinematic data obtained from this system have previously been established [16]. Moreover, improvements in motor performance for adults with spinal cord injury [17] and stroke [18] have previously been reported. To date, no previous studies have investigated the effects of the Walkbot-K system for children with CP.

Therefore, this study aimed to investigate the effects of RAGT using the Walkbot-K system on locomotor function and functional capability for daily activities in children with CP.

## 2. Methods/Design

### 2.1. Participants

We performed this study in an outpatient department of rehabilitation medicine in a tertiary university hospital from May 2018 to February 2019. Twenty children with CP (7 female and 13 male participants) were recruited and randomly assigned to two different intervention sequences. We included children with CP who had been classified as GMFCS levels II-IV (GMFCS level II (*n* = 5), III (*n* = 9), and IV (*n* = 6)). The participant’s height was restricted to 98–150 cm due to the limitations of the equipment. Children with the following characteristics were excluded: severe lower limb contractures, unhealed lower limb skin lesions, prior surgery within the past 3 months, and severe intellectual impairment resulting in an inability to understand verbal instructions.

### 2.2. Study Design

This was a single-center, single-blinded, randomized cross-over trial. Written consent was obtained after having informed the parents and patients regarding the study purpose and protocol. The hospital internal review board approved the study (IRB No. 2018-03-024) and this trial was registered at the Clinical Research Information Service (CRIS) (identifier: KCT 0003139).

**Figure 1:**
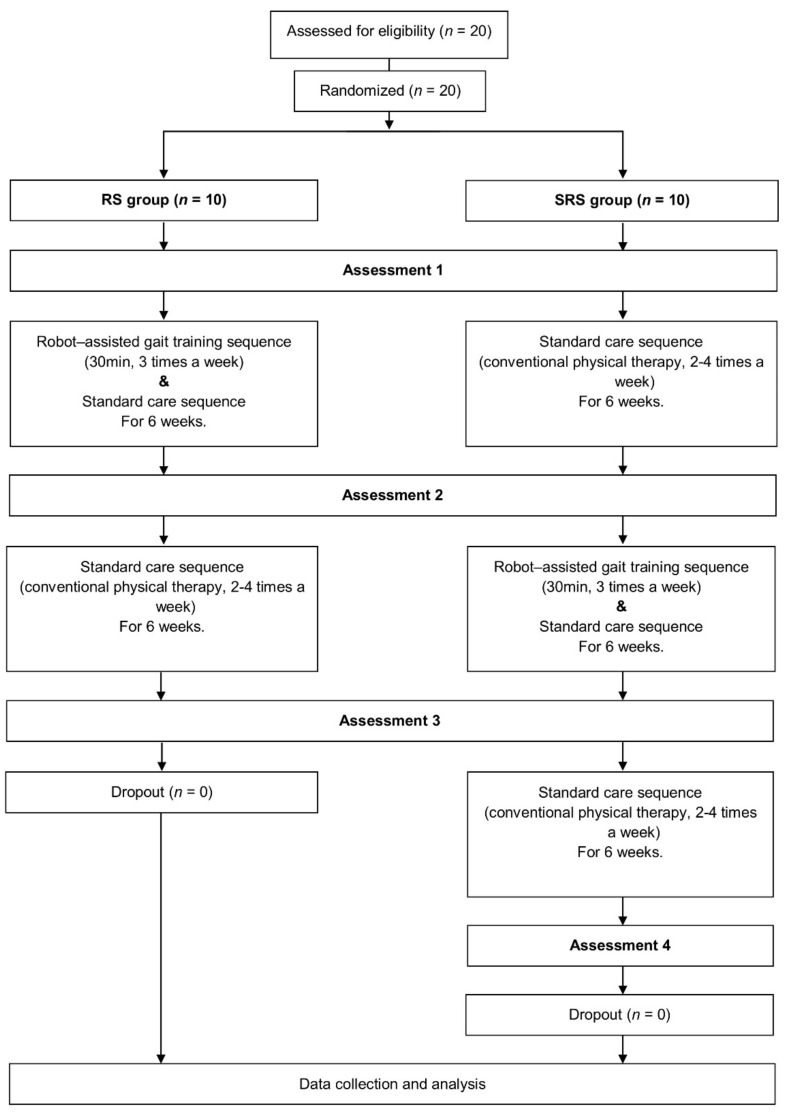
Flow diagram of the study. Abbreviations: RS, robot-assisted gait training/standard care sequence group; SRS, standard care/robot-assisted gait training/standard care sequence group.

We followed the study protocol of a previous work [19]. Two interventions, RAGT and standard care (SC), were applied for 6 weeks. The participants were randomly assigned to the RAGT/SC (RS) or an SC/RAGT/SC (SRS) groups in a 1:1 ratio. The randomization sequence was generated at the start of the trial using computer-generated sequencing. Children allocated to the RS group started with an initial assessment followed by RAGT, a subsequent second assessment, SC, and a third assessment, over a total of 12 weeks. Children allocated to the SRS group started with an initial assessment followed by SC and a second assessment. This was followed by RAGT, a third assessment, SC, and a follow-up fourth assessment, over a total of 18 weeks. The study protocol timeline is shown in Figure 1.

### 2.3. Intervention

**Figure 2:**
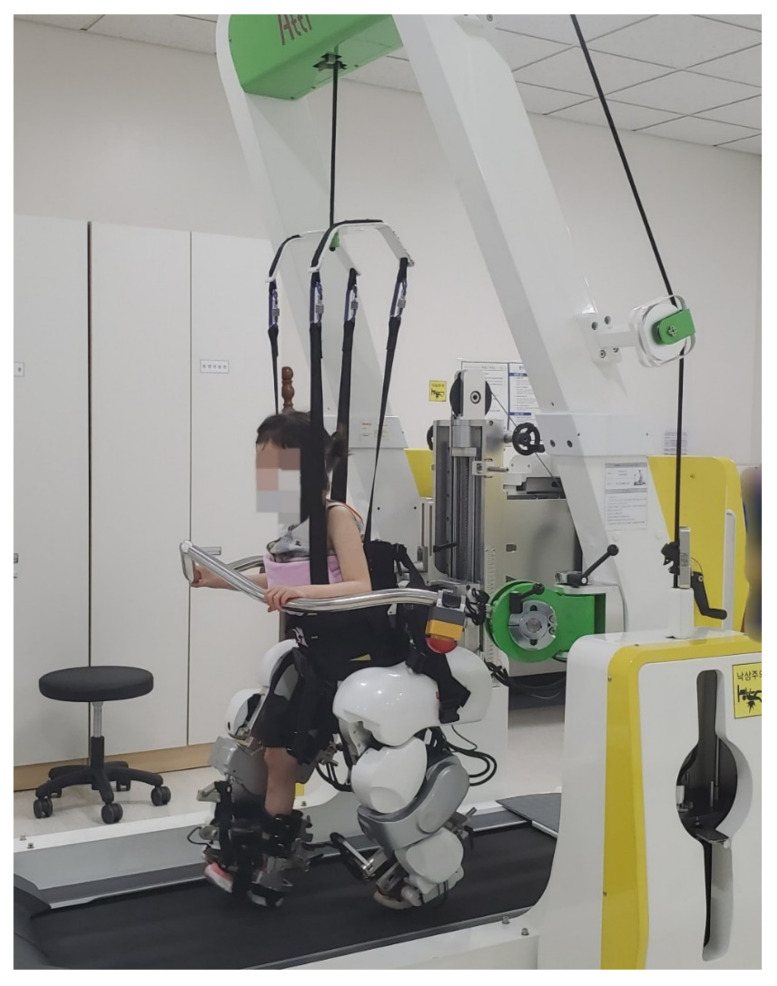
Robotic-assisted gait training using Walkbot-K.

RAGT was performed 3 times/week for 6 weeks (total, 18 sessions; minimum, 15 sessions), with a duration of 30 min on the Walkbot-K system, excluding the set-up times (Figure 2). Break time was provided when requested by the participant; however, the treatment time was maintained for at least 30 min. The Walkbot-K system is a robotic-assisted locomotor training device that has a built-in ankle actuator to provide an optimal ankle-motion trajectory during ambulation [20]. An adjustable leg length and control of the ankle joint range of motion enabled the Walkbot-K system to accurately approximate human kinematics and kinetics [16]. The Walkbot-K inclusion criteria comprised a femur length of ≥21 cm and a height of <150 cm.

Initially, RAGT BWS was set at 100%, which gradually decreased until the knees started to collapse into flexion during the stance phase. The physical therapist monitored the knee condition and controlled the BWS throughout the sessions. The walking speed was initially set at 0.5 km/h and was gradually altered until the participants self-selected a comfortable speed. The guidance force was maintained at 100% during training. One physical therapist provided all the training procedure with verbal encouragement when necessary and conventional physiotherapy continued during the intervention period.

During the SC period, the participants continued the conventional physical therapy which they had received before, including gait training, tone reduction, and balance and strengthening exercises, for 2–4 times/week for 6 weeks at the hospital. The SC intervention was different for each individual and could not be controlled; however, the participants were recommended not to change the intervention type and frequency during the study.

### 2.4. Outcome Measures

A blinded assessor who was unaware of the intervention sequence for each participant measured the study outcomes.

#### 2.4.1. Gross Motor Function Measure-88 (GMFM-88)

The GMFM-88 involves observation and quantitative evaluation to determine the degree of functional activity and gross motor function displayed by a child without considering the quality of the function. In this study, dimension C (crawling and kneeling), dimension D (standing), and dimension E (walking, running, and jumping) were assessed. The percentage scores for each dimension were used for analysis.

#### 2.4.2. Pediatric Functional Independence Measure (WeeFIM)

The WeeFIM has been reported to be useful in assessing functional independence in children with developmental disabilities and was designed to measure the influence of development strengths and difficulties in terms of independence at home, school, and in the community. A certified therapist rated each participant’s self-care (self-care and sphincter control) and mobility (transfers and locomotion) using a scale ranging from 1 to 7 (the lower the score, the greater the disability).

#### 2.4.3. Canadian Occupational Performance Measure (COPM)

The COPM was used to define five gross motor performances relevant for participation in daily life (eating, mobility, school activities, play, and indoor/outdoor games). Performance and satisfaction were rated on a scale ranging from 1 to 10 (a higher score corresponds to a better performance) at each assessment, either by a parent as a valid proxy or by the participant (if >8 years old), as applicable [10]. The mean performance and satisfaction scores for each activity were used in the analysis.

#### 2.4.4. Energy Expenditure

Energy expenditure when walking is generally expressed as two outcome measures: energy consumption (ECS) and energy cost (EC) [21]. Energy expenditure was measured using the COSMED Quark cardiopulmonary exercise testing (CPET) system (Quark CPET Gas Analyzer, COSMED^®^, Rome, Italy). The participants wore a mask for gas collection and walked 10 m independently or with physical assistance at a comfortable speed. The mean oxygen uptake (VO_2_/kg_mean_), the mean respiratory exchange ratio (RER_mean_), and walking speed values were recorded.

Gross energy consumption (ECS_gross_) was expressed in J/kg/min using the following equation: J/kg/min = (4.960 × RER_mean_ + 16.040) × VO_2_/kg [22]. Additionally, gross EC (EC_gross_), expressed in J/kg/m, was calculated through dividing ECS_gross_ with walking speed [23]. Energy expenditure was not measured when a participant was unable to walk 10 m, even with assistance.

#### 2.4.5. Body Composition

Body composition was measured in a supine position using Bioelectrical Impedance Analysis (BIA, Inbody^®^, Seoul, South Korea) with six sensors placed bilaterally on the participants’ thumbs, middle fingers, and ankles for approximately 3 min. The skeletal muscle mass (MM_skeletal_), leg muscle mass (MM_leg_), and percentage of body fat (PBF) were recorded.

### 2.5. Statistical Analysis

**Figure 3:**
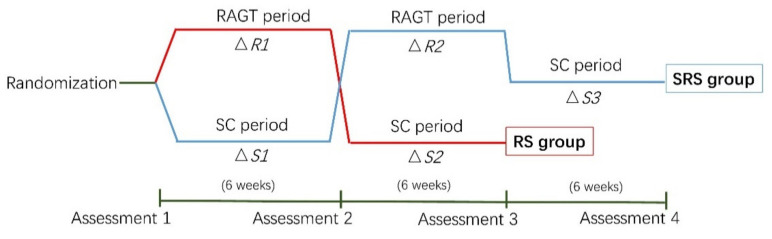
Statistical analyses. Abbreviations: RS, robot-assisted gait training/standard care sequence group; SRS, standard care/robot-assisted gait training/standard care sequence group. RAGT, robot-assisted gait training; SC, standard care; △R1, delta value during robot-assisted gait training in the RS group; △R2, delta value during robot-assisted gait training in the SRS group; △S1, delta value during the first standard care intervention in the SRS group; △S2, delta value during the standard care intervention in the RS group; △S3, delta value during the last standard care in the SRS group.

We used SPSS 25 (IBM Corporation, Armonk, NY, USA) software to perform statistical analyses. We followed the statistical analysis referenced from the protocol we referenced [19]. We analyzed the data of four effects (i.e., treatment, period, follow-up, and carry-over effects) to assess the effect of the RAGT period on GMFM-88, WeeFIM, and COPM scores. Treatment effects were compared using the delta values obtained during the RAGT and SC periods (△R1 grouped with △R2 versus △S1 grouped with △S2). The period effects were compared using the delta values obtained during the first and second training periods (△R1 grouped with △S1 versus △S2 grouped with △R2). The follow-up effects were compared as follows: second assessment of the RS group grouped with third of SRS group versus third assessment of RS group grouped with fourth of SRS group. Additionally, the carry-over effects were analyzed using the delta value during all periods for the RS group versus the former two periods of the SRS group (△R1 grouped with △S2 versus △R2 grouped with △S1) (Figure 3). Between-group differences at baseline and the carry-over effects were analyzed using independent *t*- or Mann–Whitney U tests for continuous variables and chi-square or Fisher’s exact tests for binary variables, as appropriate. The paired *t*- or Wilcoxon’s signed-rank tests were used to analyze the treatment, period, and follow-up effects and to analyze data concerning body composition and energy expenditure, respectively. Factors associated with functional outcomes were measured according to the age (≤6 years versus >6 years) and GMFCS levels (levels II–III (ambulatory) vs. level IV (non-ambulatory)). Finally, a Mann–Whitney U test was used to determine the between-group differences. The level of statistical significance for all tests was set at *p* < 0.05.

## 3. Results

Twenty participants completed the study. Especially, 10 and 10 children were assigned to the RS (age range, 3–10 years) and RSR (age range, 4–11 years) groups, respectively. There were no significant differences in age, height, weight, type of CP, and GMFCS level distribution between the RS and SRS groups (Table 1). The RAGT parameters did not significantly differ between the two groups (Table 2). Similarly, the baseline parameters for GMFM, WeeFIM, and COPM between the two groups also did not differ significantly (Table 3). After performing all the sessions, the BWS and walking speed gradually decreased or increased to a mean of 49% and 1.28 km/h, respectively. No safety issues were reported and none of the participants experienced any side-effects during RAGT.

### 3.1. GMFM-88

**Figure 4:**
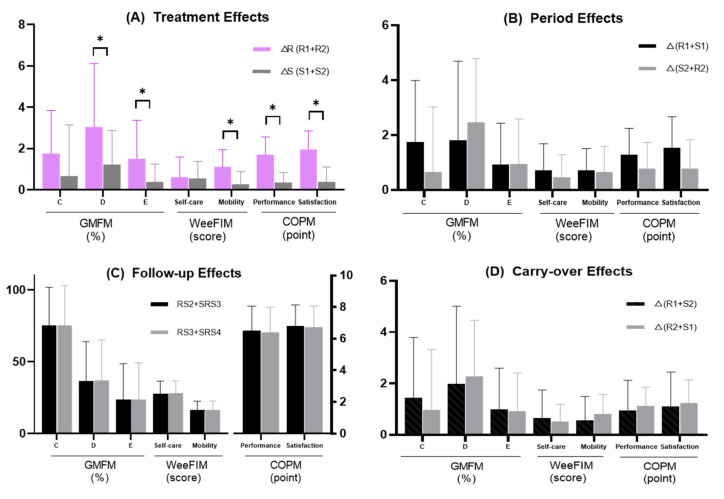
RAGT showed significant treatment effects in the GMFM D and E, WeeFIM mobility subtotal, and COPM performance and satisfaction scores. The period, carry-over, and follow-up effects did not show statistical significance. (**A**) Treatment effects; (**B**) period effects; (**C**) follow-up effects; (**D**) carry-over effects. Abbreviations: GMFM-88, gross motor function measure-88; GMFM C, gross motor function measure dimension C; GMFM D, gross motor function measure dimension D; GMFM E, gross motor function measure dimension E; WeeFIM, pediatric functional independence measure; COPM, Canadian occupational performance measure; RS, robot-assisted gait training/standard care sequence group; SRS, standard care/robot-assisted gait training/standard care sequence group; △R1, change during robot-assisted gait training in the RS group; △R2, change during robot-assisted gait training in the SRS group; △S1, change during first standard care in the SRS group; △S2, change during standard care in the RS group; RS2, assessment 2 in the RS group; RS3, assessment 3 in the RS group; SRS3, assessment 3 in the SRS group; SRS4, assessment 4 in the SRS group. * *p* < 0.05 by paired *t*-test or Wilcoxon signed-rank test.

The RAGT period showed significant treatment effects in relation to GMFM D (*p* = 0.018) and E (*p* = 0.021) scores, with both scores being significantly improved during the RAGT period compared with those in the SC period. However, the change in the GMFM C score was not significantly different between the RAGT and SC periods (*p* = 0.093) (Figure 4). The period, follow-up, and carry-over effects of the GMFM C, D, and E scores were not statistically significant (Figure 4).

### 3.2. WeeFIM

There was a significant treatment effect in terms of the mobility subtotal score of the WeeFIM (*p* = 0.007), which significantly improved during the RAGT period compared with that in the SC period. The treatment effect of the self-care subtotal and the period, follow-up, and carry-over effects of WeeFIM self-care and mobility subtotals were not statistically significant (Figure 4).

### 3.3. COPM

The performance and satisfaction scores of COPM indicated a significant treatment effect (performance, *p* < 0.001; satisfaction, *p* = 0.001); however, the period, follow-up, and carry-over effects were not statistically significant (Figure 4).

### 3.4. Energy Expenditure

Energy expenditure was measured in 14 participants. Five children (GMFCS level IV) who could not walk and one child (GMFCS level III) unable to fit the test mask due to a small face size were not tested. The EC_gross_ significantly decreased at post-RAGT assessment compared to pre-RAGT (*p* = 0.041). No significant differences were found in terms of VO_2_/kg_mean_, gait speed, RER_mean,_ and ECS_gross_ (Table 4).

### 3.5. Body Composition

Body compositions were measured in 19 participants (one patient was excluded due to poor participation). The MM_skeletal_ significantly increased at post-RAGT assessment compared to pre-RAGT (*p* = 0.014). The changes in the MM_leg_ and PBF values were not statistically significant but both measures showed an improving trend (Table 4).

### 3.6. Factors Associated with Functional Outcome

We investigated the association between factors such as age and GMFCS level with the delta value of significantly improved outcomes in terms of treatment effects (i.e., GMFM D, GMFM E, mobility, performance, and satisfaction). Significant between-level improvements in the GMFM D scores were mainly observed in participants classified as GMFCS levels II–III rather than in those classified as level IV (*p* = 0.038) (Table 5).

## 4. Discussion

In this study, gross motor function and functional capability for daily activities, measured using GMFM-88 and using WeeFIM and COPM, respectively, improved after 6 weeks of RAGT. Two different sequences were designed in this randomized cross-over trial to investigate the effects of the Walkbot-K RAGT in children with CP. Our research protocol was based on that of a previous study and the strength of its design was that each child received both forms of treatment [19]. Therefore, the participants had their own control and only the half the number of patients was needed compared with what would have been required if we used a parallel-group design. The report’s authors, whose protocol we referenced, published their results that they stopped the trial earlier than planned because of recruitment issues [15]. Despite some recruitment difficulties, our 20 participants completed at least 15 of 18 RAGT sessions without dropping out.

In previous studies, the number of RAGT sessions varied from 10 to 40 [11,12,13,24], and in a recent RCT that reported positive results [9,11], the number of therapy sessions ranged from 18 to 20. Study durations also ranged from 2 to 10 weeks in previous reports [13,24]. Interestingly, an optimal study duration is likely to be from 4 to 6 weeks as positive results have been observed in most studies having this duration [9,11,25]. In our study, 18 RAGT sessions were performed for 6 weeks, during which positive outcomes were observed.

The participants in our study were younger (age range, 3–11 years; mean, 6.75 years) and more severely affected (mean GMFCS level, 3.05) than those in most previous RAGT studies [8,11,13,15]. The younger age limited the length of the lower limb, which influenced training speed and total training distance. Therefore, the training speed and total distance covered in our study were also lower and the BWS percentage was found to be higher than that observed in most previous studies [9,13]. The guidance force was not changed during the training sessions and remained at 100% because study participants with lower limb weakness would not have been able to continue training if the guidance force had been reduced.

Improvements in GMFM D and E scores post-RAGT have also been reported in several previous studies [8,9,10,26,27], whereas other studies have reported no change or a slight change without significance [11,15]. A recent study suggested that an average GMFM D score change in score of 1.2 could be used as a reference datum for the minimal clinical important difference (MCID) score [28]. In our study, the GMFM D score increased (average, 3.0) higher than the MCID score post-RAGT. The change in the GMFM D score in our study was higher than that reported in previous studies, which included participants who had similar ages and milder impairments compared to our study participants and who received 40 sessions of RAGT only or combined with task-oriented physiotherapy [24]. The factor analysis in our study showed more improvements in the GMFM D score in ambulatory compared with non-ambulatory children with CP. A similar analysis was performed in a previous study, in which the participants were divided into those having GMFCS levels I–II and those having levels III–IV [26]. The results showed that the severity of motor impairment affected the amount of achieved improvement. However, another study indicated that participants with GMFCS level IV experienced significant improvement in walking-related outcomes compared with those classified as GMFCS level II or III post-RAGT [29]. In one study, the MCID of GMFM E was reported to be 1.2 and involved only participants with GMFCS levels I, II, and III [30], whereas another recent study showed that the MCID was 0.3 in participants with GMFCS levels I–IV [28]. Our study findings showed a gain improvement in GMFM E scores of 1.5 post-RAGT, which was higher than the MCID reported in one study involving mildly impaired patients with CP [30].

The mobility of WeeFIM also significantly increased post-RAGT in our study. Few studies have investigated the influence of RAGT on the WeeFIM outcomes [13,29]. One retrospective study showed similar results to those of our study, as the mobility subtotal and total WeeFIM scores increased by approximately one and three points, respectively, in participants with GMFCS levels II–IV post-RAGT [29]. Different to the GMFM, which assesses displayed function, the WeeFIM mobility score can also be used to measure functional capabilities in daily activities. However, one RCT study reported negative WeeFIM total score results post-RAGT [13]. Therefore, further research is required to determine the influence of RAGT in WeeFIM.

We investigated improvements in participation using the COPM outcomes. Significant changes in participation and satisfaction (1.71 and 1.94, respectively) in terms of treatment effects were observed in our study. Only two previous studies using participation measures involving COPM have also reported a significant increase in COPM post-RAGT but one was a prospective controlled cohort study and the other was a case report [10,31]. Several reports have attributed the limited efficacy of RAGT to the lack of engaging activities during training [32,33], and COPM has been reported to be a feasible option for determining outcome measures in a pediatric rehabilitation research [34]. However, to date, there is insufficient evidence to support the influence of COPM post-RAGT. Although the COPM findings in our study were lower than the MCID (two points, for performance or satisfaction) [35], our positive results could serve as a preliminary support for future research.

Aras et al. [12] reported that GMFM D and E improvements lasted 3 months following 5 weeks of robot training. Moreover, COPM improvements following 12 RAGT sessions were observed at the 8-week follow-up [10]. In our study, no significant follow-up or carry-over effects were observed at the 6-week follow-up.

Treatment effects in our study showed significant improvement during the RAGT period; however, SC intervention was continued during the RAGT period, with no control regarding the type or frequency of interventions. Thereby, the aforementioned improvement may be also related to multi-modality intervention. The efficiency of RAGT combined with physical therapy is displayed in the improved GMFM score in children with CP classified as GMFCS level III status in a previous study; an uncontrolled study also showed improvement of GMFM C, D, and E when RAGT combined with conventional rehabilitation was performed [15,36]. However, the GMFM score also improved in children who received only RAGT compared to that in children who received conventional rehabilitation group in a previous RCT study [8]. Further analyses are needed to confirm the effectiveness of performing only RAGT using the Walkbot system in children with CP.

High EC is a factor that contributes to reduced integration into the activities of daily living among children with CP due to abnormal energy motor responses and muscle activity [22]. EC in children with CP can be up to three times higher than EC in typically developing (TD) peers [37,38], equivalent to intense exercise while merely walking at a comfortable speed [39]. Previous studies have investigated several approaches that aimed to reduce metabolic demand in children with CP, such as lifestyle intervention [40] and ankle exoskeleton assistance [41]. Another study investigated the influence of EC post-RAGT in children with CP and reported a reduction in EC following 20 RAGT sessions compared with partial BWS training [12]. This improvement in walking economy enabled children with CP to participate in more activities of daily living, which may explain the trend of increased COPM score in our study. However, in our study, EC was evaluated by self-comparison without including a control group, and therefore, further research is required to confirm the association between RAGT effectiveness and EC.

Gait speed slightly increased post-RAGT but this increase was not statistically significant. Our gait speed results were consistent with those of a previous controlled study [11,13]. Most studies involving RAGT have shown an increase in gait speed after training [8,36] but we were not able to eliminate the possible influence of the task mask connected to the CPET device worn by participants during gait speed measurement.

Previous studies have reported that the muscle mass in individuals with CP was lower than that in TD individuals [42]. The skeletal muscle plays an important role in daily life as it is the largest tissue component of the lean body mass in humans [43]. MM_skeletal_ is crucial for movement and balance [44] and is positively correlated with gross motor function in children with CP [42,45]. Most previous studies have evaluated muscle strength or muscle size after training using magnetic resonance imaging (MRI) [46] or dynamometry [47]. In our study, MM_skeletal_ significantly increased post-RAGT but the MM_leg_ increases were not found to be significant. No significant change in PBF was observed; however, the mean percentage post-RAGT decreased and was close to that of TD peers (mean PBF in TD peers, 23%) [42].

## 5. Study Limitations

We designed this study as a cross-over trial to present higher precision with small sample sizes. However, the cyclic 6-week duration could not exclude a degree of maturation to a certain extent. The participants received RAGT combined with SC in the RAGT period and did not receive additional treatment except for SC during the SC period; therefore, the possibility of an extra 30 min of training affecting our study results cannot be eliminated. As the provider of the protocol suggested [19], we did not introduce a washout period to reduce the load of the participants to a minimum. Although we considered the period and carry-over effects to exclude the influence of the former type of intervention on the subsequent one, this might have influenced our results, as the improvements were just related to the RAGT. Five participants who were classified as GMFCS level IV were excluded from the EC measurements because they could not conduct the oxygen uptake and walking speed tests during exercise. Therefore, the EC results in our study need to be interpreted with caution, except for the results concerning participants classified as GMFCS levels II–III. Considering most of the interventions that measured muscle strength and rarely used MRI or other approaches to focus on the muscle volume, caution should be taken in terms of whether the BIA data used to measure body composition could be considered representative of the general population. Moreover, to reduce the assessment time and avoid the risk of some participants declining to continue with treatment, energy expenditure and body composition were analyzed only pre- and post-RAGT; therefore, it was difficult to determine whether the extent of changes observed was due to RAGT only.

## 6. Conclusions

To our knowledge, this study is the first to investigate the efficiency of the Walkbot-K RAGT in children with CP using a randomized cross-over study design. Especially, we showed improvement in locomotor function and functional capability of participants when performing daily activities. These effects were better in the ambulatory children with CP. However, as SC intervention was continued during the RAGT period, these improvements may be also related to multiple treatment effects. Further research is required using a higher level of trial design and more participant numbers to confirm the effectiveness and the correlation in different age-stages and types of children with CP.

## Figures and Tables

**Table 1:** Demographic characteristics of the participants.

Group	Total	RS (*n* = 10)	SRS (*n* = 10)	*p*-Value *
Age (years)	6.75 (2.15)	6.0 (2.21)	7.5 (1.90)	0.121
Sex, *n*				
Female	7	3	4	1.000
Male	13	7	6	
Height (cm)	113.91 (13.67)	110.92 (12.34)	116.89 (14.91)	0.342
Weight (kg)	22.23 (8.81)	21.10 (9.24)	23.36 (8.69)	0.545
GMFCS level, *n*				
II	5	4	1	0.284
II	9	3	6	
IV	6	3	3	
Tone abnormality				
Spastic	17	8	9	1.000
Dyskinetic	1	1	0	
Mixed	2	1	1	
Body involvement				
Unilateral	1	1	0	1.000
Bilateral	19	9	10	

Values are presented as means (standard deviation). Abbreviations: RS, robot-assisted gait training/standard care sequence group; SRS, standard care/robot-assisted gait training/standard care sequence group; CP, cerebral palsy; GMFCS, gross motor function classification system. * *p*-value by *t*-test or Mann–Whitney *U* test (for continuous variables) or Fisher’s exact test (for categorical variables).

**Table 2:** Robot training parameter.

	Total	RS Group	SRS Group	*p-*Value *
Gait Speed (km/h)	1.28 (0.16)	1.30 (0.17)	1.25 (0.14)	0.507
Body-weight support (%)	49.0 (7.18)	49.0 (7.38)	49.0 (7.38)	1.000
Total sessions	16.10 (1.17)	16.50 (1.08)	15.70 (1.16)	0.092
Total distance (km)	11.16 (2.26)	11.51 (2.07)	10.80 (2.48)	0.494

Values are presented as means (standard deviation). Abbreviations: RS, robot-assisted gait training/standard care sequence group; SRS, standard care/robot-assisted gait training/standard care sequence group. * *p*-value by *t*-test or Mann-Whitney *U* test.

**Table 3:** Baseline functional characteristics of the participants.

		RS Group	SRS Group	*p-*Value *
GMFM-88				
	C (%)	77.86 (19.54)	70.60 (26.94)	0.544
	D (%)	41.28 (31.58)	26.27 (19.99)	0.250
	E (%)	30.77 (27.96)	12.81 (11.66)	0.172
WeeFIM				
	Self-care	25.80 (6.25)	27.60 (10.27)	0.641
	Mobility	14.10 (4.72)	15.80 (6.94)	0.530
COPM				
	Performance	4.88 (1.48)	4.00 (0.89)	0.125
	Satisfaction	4.88 (1.54)	3.98 (0.94)	0.133

Values are presented as means (standard deviation). Abbreviations: RS, robot-assisted gait training/standard care sequence group; SRS, standard care/robot-assisted gait training/standard care sequence group. GMFM-88, gross motor function measure-88; GMFM C, gross motor function measure dimension C; GMFM D, gross motor function measure dimension D; GMFM E, gross motor function measure dimension E; WeeFIM, pediatric functional independence measure; COPM, Canadian occupational performance measure. * *p*-value by *t-*test or Mann–Whitney U test.

**Table 4:** Effect of RAGT on energy expenditure and body composition.

Pre RAGT	Post RAGT	*p*-Value	
Energy expenditure	(*n* = 14)	(*n* = 14)	
Oxygen uptake_mean_ (mL/min/kg)	17.25 (6.02)	16.01 (3.66)	0.485
Speed (m/min)	20.61 (7.35)	21.68 (7.18)	0.223
RER_mean_	0.99 (0.15)	0.96 (0.10)	0.531
ECS_gross_ (J/kg/min)	359.21 (118.34)	332.89 (76.98)	0.311
EC_gross_ (J/kg/m)	20.34 (11.35)	17.17 (7.03)	0.041 *
Body composition	(*n* = 19)	(*n* = 19)	
MM_skeletal_ (kg)	8.75 (4.67)	9.01 (4.63)	0.014 *
MM_leg_ (kg)	4.20 (2.66)	4.34 (2.75)	0.103
PBF (%)	26.04 (9.87)	23.24 (12.64)	0.085

Values are presented as means (standard deviation). Abbreviations: RAGT, robot-assisted gait training; RER_mean_, mean respiratory exchange ratio; ECS_gross_, gross energy consumption; EC_gross_, gross energy cost; MM_skeletal_, skeletal muscle mass; MM_leg_, leg muscle mass; PBF, percentage of body fat. * *p* < 0.05 by paired *t*-test or Wilcoxon signed-rank test.

**Table 5:** The changes of functional outcome measures according to age and GMFCS level.

	Age			GMFCS Level		
	Age 6 (*n* = 9)	Age > 6 (*n* = 11)	*p-*Value	Level II-III (*n* = 14)	Level IV (*n* = 6)	*p-*Value
GMFM						
D	2.79 (3.51)	3.26 (2.83)	0.871	3.81 (2.82)	1.28 (3.14)	0.038 *
E	1.35 (1.97)	1.82 (1.76)	0.508	2.06 (1.96)	0.57 (0.90)	0.108
WeeFIM						
Mobility	1.00 (1.00)	1.18 (0.75)	0.491	7.80 (4.96)	3.14 (3.02)	0.135
COPM						
Performance	1.69 (1.08)	1.73 (0.66)	0.909	1.80 (0.76)	1.50 (1.08)	0.360
Satisfaction	1.91 (1.03)	1.96 (0.84)	0.939	1.93 (0.88)	1.97 (1.05)	0.934

Values are presented as means (standard deviation). Abbreviations: GMFCS, gross motor function classification system; RAGT, robot-assisted gait training; GMFM D, gross motor function measure dimension D; GMFM E, gross motor function measure dimension E; WeeFIM, pediatric functional independence measure; COPM, Canadian occupational performance measure; * *p* < 0.05 by Mann–Whitney U test.

## References

[1] Rosenbaum P., Paneth N., Leviton A., Goldstein M., Bax M., Damiano D., Dan B., Jacobsson B. (2007). A report: The definition and classification of cerebral palsy April 2006. Dev. Med. Child Neurol. Suppl..

[2] Imms C. (2008). Children with cerebral palsy participate: A review of the literature. Disabil. Rehabil..

[3] Van Wely L., Balemans A.C., Becher J.G., Dallmeijer A.J. (2014). Physical activity stimulation program for children with cerebral palsy did not improve physical activity: A randomised trial. J. Physiother.

[4] Lefmann S., Russo R., Hillier S. (2017). The effectiveness of robotic-assisted gait training for paediatric gait disorders: Systematic review. J. Neuroeng. Rehabil..

[5] Carvalho I., Pinto S.M., Chagas D.D.V., Praxedes Dos Santos J.L., de Sousa Oliveira T., Batista L.A. (2017). Robotic Gait Training for Individuals With Cerebral Palsy: A Systematic Review and Meta-Analysis. Arch. Phys. Med. Rehabil..

[6] Lefeber N., Swinnen E., Kerckhofs E. (2017). The immediate effects of robot-assistance on energy consumption and cardiorespiratory load during walking compared to walking without robot-assistance: A systematic review. Disabil. Rehabil. Assist. Technol..

[7] Beretta E., Storm F.A., Strazzer S., Frascarelli F., Petrarca M., Colazza A., Cordone G., Biffi E., Morganti R., Maghini C. (2020). Effect of Robot-Assisted Gait Training in a Large Population of Children with Motor Impairment Due to Cerebral Palsy or Acquired Brain Injury. Arch. Phys. Med. Rehabil..

[8] Wallard L., Dietrich G., Kerlirzin Y., Bredin J. (2018). Effect of robotic-assisted gait rehabilitation on dynamic equilibrium control in the gait of children with cerebral palsy. Gait Posture.

[9] Wallard L., Dietrich G., Kerlirzin Y., Bredin J. (2017). Robotic-assisted gait training improves walking abilities in diplegic children with cerebral palsy. Eur. J. Paediatr. Neurol..

[10] Schroeder A.S., Homburg M., Warken B., Auffermann H., Koerte I., Berweck S., Jahn K., Heinen F., Borggraefe I. (2014). Prospective controlled cohort study to evaluate changes of function, activity and participation in patients with bilateral spastic cerebral palsy after Robot-enhanced repetitive treadmill therapy. Eur. J. Paediatr. Neurol..

[11] Wu M., Kim J., Arora P., Gaebler-Spira D.J., Zhang Y. (2017). Effects of the Integration of Dynamic Weight Shifting Training Into Treadmill Training on Walking Function of Children with Cerebral Palsy: A Randomized Controlled Study. Am. J. Phys. Med. Rehabil..

[12] Aras B., Yasar E., Kesikburun S., Turker D., Tok F., Yilmaz B. (2019). Comparison of the effectiveness of partial body weight-supported treadmill exercises, robotic-assisted treadmill exercises, and anti-gravity treadmill exercises in spastic cerebral palsy. Turk. J. Phys. Med. Rehabil..

[13] Smania N., Bonetti P., Gandolfi M., Cosentino A., Waldner A., Hesse S., Werner C., Bisoffi G., Geroin C., Munari D. (2011). Improved gait after repetitive locomotor training in children with cerebral palsy. Am. J. Phys. Med. Rehabil..

[14] Druzbicki M., Rusek W., Snela S., Dudek J., Szczepanik M., Zak E., Durmala J., Czernuszenko A., Bonikowski M., Sobota G. (2013). Functional effects of robotic-assisted locomotor treadmill thearapy in children with cerebral palsy. J. Rehabil. Med..

[15] Ammann-Reiffer C., Bastiaenen C.H.G., Meyer-Heim A.D., van Hedel H.J.A. (2020). Lessons learned from conducting a pragmatic, randomized, crossover trial on robot-assisted gait training in children with cerebral palsy (PeLoGAIT). J. Pediatr. Rehabil. Med..

[16] Lee D.R., Shin Y.K., Park J.-H., You J.H. (2016). Concurrent Validity and Test-Retest Reliability of the Walkbot-K System for Robotic Gait Training. J. Mech. Med. Biol..

[17] Hwang J., Shin Y., Park J.H., Cha Y.J., You J.S.H. (2018). Effects of Walkbot gait training on kinematics, kinetics, and clinical gait function in paraplegia and quadriplegia. NeuroRehabilitation.

[18] Kim S.Y., Yang L., Park I.J., Kim E.J., JoshuaPark M.S., You S.H., Kim Y.H., Ko H.Y., Shin Y.I. (2015). Effects of Innovative WALKBOT Robotic-Assisted Locomotor Training on Balance and Gait Recovery in Hemiparetic Stroke: A Prospective, Randomized, Experimenter Blinded Case Control Study With a Four-Week Follow-Up. IEEE Trans. Neural Syst. Rehabil. Eng..

[19] Ammann-Reiffer C., Bastiaenen C.H., Meyer-Heim A.D., van Hedel H.J. (2017). Effectiveness of robot-assisted gait training in children with cerebral palsy: A bicenter, pragmatic, randomized, cross-over trial (PeLoGAIT). BMC Pediatr..

[20] Jung J.H., Lee N.G., You J.H., Lee D.C. (2009). Validity and feasibility of intelligent Walkbot system. Electron. Lett..

[21] Brehm M.A., Knol D.L., Harlaar J. (2008). Methodological considerations for improving the reproducibility of walking efficiency outcomes in clinical gait studies. Gait Posture.

[22] Ijmker T., Houdijk H., Lamoth C.J., Beek P.J., van der Woude L.H. (2013). Energy cost of balance control during walking decreases with external stabilizer stiffness independent of walking speed. J. Biomech..

[23] de Groot J.F., Takken T., van Brussel M., Gooskens R., Schoenmakers M., Versteeg C., Vanhees L., Helders P. (2011). Randomized controlled study of home-based treadmill training for ambulatory children with spina bifida. Neurorehabil. Neural Repair.

[24] Peri E., Turconi A.C., Biffi E., Maghini C., Panzeri D., Morganti R., Pedrocchi A., Gagliardi C. (2017). Effects of dose and duration of Robot-Assisted Gait Training on walking ability of children affected by cerebral palsy. Technol. Health Care.

[25] Meyer-Heim A., Ammann-Reiffer C., Schmartz A., Schafer J., Sennhauser F.H., Heinen F., Knecht B., Dabrowski E., Borggraefe I. (2009). Improvement of walking abilities after robotic-assisted locomotion training in children with cerebral palsy. Arch. Dis. Child..

[26] Borggraefe I., Schaefer J.S., Klaiber M., Dabrowski E., Ammann-Reiffer C., Knecht B., Berweck S., Heinen F., Meyer-Heim A. (2010). Robotic-assisted treadmill therapy improves walking and standing performance in children and adolescents with cerebral palsy. Eur. J. Paediatr. Neurol..

[27] Meyer-Heim A., Borggraefe I., Ammann-Reiffer C., Berweck S., Sennhauser F.H., Colombo G., Knecht B., Heinen F. (2007). Feasibility of robotic-assisted locomotor training in children with central gait impairment. Dev. Med. Child Neurol..

[28] Storm F.A., Petrarca M., Beretta E., Strazzer S., Piccinini L., Maghini C., Panzeri D., Corbetta C., Morganti R., Reni G. (2020). Minimum Clinically Important Difference of Gross Motor Function and Gait Endurance in Children with Motor Impairment: A Comparison of Distribution-Based Approaches. Biomed. Res. Int..

[29] van Hedel H.J., Meyer-Heim A., Rusch-Bohtz C. (2016). Robot-assisted gait training might be beneficial for more severely affected children with cerebral palsy. Dev. Neurorehabil..

[30] Oeffinger D., Bagley A., Rogers S., Gorton G., Kryscio R., Abel M., Damiano D., Barnes D., Tylkowski C. (2008). Outcome tools used for ambulatory children with cerebral palsy: Responsiveness and minimum clinically important differences. Dev. Med. Child Neurol..

[31] Kuroda M., Nakagawa S., Mutsuzaki H., Mataki Y., Yoshikawa K., Takahashi K., Nakayama T., Iwasaki N. (2020). Robot-assisted gait training using a very small-sized Hybrid Assistive Limb(R) for pediatric cerebral palsy: A case report. Brain Dev..

[32] Swinnen E., Duerinck S., Baeyens J.P., Meeusen R., Kerckhofs E. (2010). Effectiveness of robot-assisted gait training in persons with spinal cord injury: A systematic review. J. Rehabil. Med..

[33] Ada L., Dean C.M., Vargas J., Ennis S. (2010). Mechanically assisted walking with body weight support results in more independent walking than assisted overground walking in non-ambulatory patients early after stroke: A systematic review. J. Physiother.

[34] Cusick A., McIntyre S., Novak I., Lannin N., Lowe K. (2006). A comparison of goal attainment scaling and the Canadian Occupational Performance Measure for paediatric rehabilitation research. Pediatr. Rehabil..

[35] Eyssen I.C., Steultjens M.P., Oud T.A., Bolt E.M., Maasdam A., Dekker J. (2011). Responsiveness of the Canadian occupational performance measure. J. Rehabil. Res. Dev..

[36] Matsuda M., Iwasaki N., Mataki Y., Mutsuzaki H., Yoshikawa K., Takahashi K., Enomoto K., Sano K., Kubota A., Nakayama T. (2018). Robot-assisted training using Hybrid Assistive Limb(R) for cerebral palsy. Brain Dev..

[37] Johnston T.E., Moore S.E., Quinn L.T., Smith B.T. (2004). Energy cost of walking in children with cerebral palsy: Relation to the Gross Motor Function Classification System. Dev. Med. Child Neurol..

[38] Brehm M.A., Becher J., Harlaar J. (2007). Reproducibility evaluation of gross and net walking efficiency in children with cerebral palsy. Dev. Med. Child Neurol..

[39] Balemans A.C., Bolster E.A., Brehm M.A., Dallmeijer A.J. (2017). Physical Strain: A New Perspective on Walking in Cerebral Palsy. Arch. Phys. Med. Rehabil..

[40] Slaman J., Roebroeck M., van der Slot W., Twisk J., Wensink A., Stam H., van den Berg-Emons R., Group L.M.R. (2014). Can a lifestyle intervention improve physical fitness in adolescents and young adults with spastic cerebral palsy? A randomized controlled trial. Arch. Phys. Med. Rehabil..

[41] Orekhov G., Fang Y., Luque J., Lerner Z.F. (2020). Ankle Exoskeleton Assistance Can Improve Over-Ground Walking Economy in Individuals With Cerebral Palsy. IEEE Trans. Neural Syst. Rehabil. Eng..

[42] Walker J.L., Bell K.L., Stevenson R.D., Weir K.A., Boyd R.N., Davies P.S. (2015). Differences in body composition according to functional ability in preschool-aged children with cerebral palsy. Clin. Nutr..

[43] Zhang C., Whitney D.G., Singh H., Slade J.M., Shen Y., Miller F., Modlesky C.M. (2019). Statistical Models to Assess Leg Muscle Mass in Ambulatory Children With Spastic Cerebral Palsy Using Dual-Energy X-Ray Absorptiometry. J. Clin. Densitom..

[44] Szulc P., Beck T.J., Marchand F., Delmas P.D. (2005). Low skeletal muscle mass is associated with poor structural parameters of bone and impaired balance in elderly men--The MINOS study. J. Bone Miner. Res..

[45] Duran I., Schutz F., Hamacher S., Semler O., Stark C., Schulze J., Rittweger J., Schoenau E. (2017). The functional muscle-bone unit in children with cerebral palsy. Osteoporos. Int..

[46] Gillett J.G., Lichtwark G.A., Boyd R.N., Barber L.A. (2018). Functional Anaerobic and Strength Training in Young Adults with Cerebral Palsy. Med. Sci. Sports Exerc..

[47] Bayon C., Martin-Lorenzo T., Moral-Saiz B., Ramirez O., Perez-Somarriba A., Lerma-Lara S., Martinez I., Rocon E. (2018). A robot-based gait training therapy for pediatric population with cerebral palsy: Goal setting, proposal and preliminary clinical implementation. J. Neuroeng. Rehabil..

